# First Case of Glyphosate Resistance in *Bromus catharticus* Vahl.: Examination of Endowing Resistance Mechanisms

**DOI:** 10.3389/fpls.2021.617945

**Published:** 2021-02-18

**Authors:** Marcos Yanniccari, José G. Vázquez-García, María E. Gómez-Lobato, Antonia M. Rojano-Delgado, Pedro L. da C. A. Alves, Rafael De Prado

**Affiliations:** ^1^National Scientific and Technical Research Council, Buenos Aires, Argentina; ^2^Chacra Experimental Integrada Barrow, MDA-INTA, Tres Arroyos, Argentina; ^3^Department of Agricultural Chemistry and Edaphology, University of Cordoba, Cordoba, Spain; ^4^Plant Physiology Institute (INFIVE), National University of La Plata, La Plata, Argentina; ^5^School of Agricultural and Veterinarian Sciences, Sâo Paulo State University (Unesp), Jaboticabal, Brazil

**Keywords:** Brome, EPSPS gene, shikimate, glyphosate absorption, glyphosate translocation

## Abstract

*Bromus catharticus* Vahl. has been used as a valuable forage crop, but it has also been noted as a weed of winter crops and an invader in several countries. In Argentina, a putative glyphosate-resistant population of *B. catharticus* was identified as a consequence of the lack of effective control with glyphosate in the pre-sowing of wheat. Plant survival and shikimate accumulation analysis demonstrated a lower glyphosate-sensitivity of this population in comparison to a susceptible *B. catharticus* population. The resistant population was 4-fold more resistant to glyphosate than its susceptible counterpart. There was no evidence of target-site mechanisms of glyphosate resistance or an enhanced capacity to metabolize glyphosate in the resistant population. However, the resistant plants showed a lower foliar retention of glyphosate (138.34 μl solution g^−1^ dry weight vs. 390.79 μl solution g^−1^ dry weight), a reduced absorption of ^14^C-glyphosate (54.18 vs. 73.56%) and lower translocation of ^14^C-glyphosate from the labeled leaf (27.70 vs. 62.36%). As a result, susceptible plants accumulated a 4.1-fold higher concentration of ^14^C-glyphosate in the roots compared to resistant plants. The current work describes the first worldwide case of glyphosate resistance in *B. catharticus*. A reduced foliar retention of herbicide, a differential rate of glyphosate entry into leaves and an altered glyphosate translocation pattern would be the most likely mechanisms of glyphosate exclusion.

## Introduction

The genus *Bromus* L. comprises approximately 150 species distributed across temperate and cool regions of both hemispheres (Planchuelo and Peterson, 2000). Several species are used as natural pasture for grazing or have been introduced as forage in different countries (Planchuelo and Peterson, 2000). However, some *Bromus* species are aggressive invaders posing enormous threats to native ecosystems (Bradford and Lauenroth, 2006; Speziale et al., 2014; Atkinson and Brown, 2016) or troublesome weeds on arable lands (Cussans et al., 1994; Andersson et al., 2002; Kleemann and Gill, 2006). Among them, *Bromus catharticus* Vahl. has been used as a valuable forage crop, but it has also been identified as a weed of winter crops and an invader in several countries (Casha et al., 2011; Ahumada and Troiani, 2016; Kovář, 2018).

*Bromus catharticus* originated in the Pampas of South America and was widely introduced into temperate regions worldwide (Planchuelo and Peterson, 2000; Planchuelo, 2006), but it also escaped into the wild in four continents (Dastgheib et al., 2003; Di Tomaso and Healy, 2007; Verloove, 2012; Muzafar et al., 2016; Bromilow, 2018). This is an annual, biennial, or perennial species and it shows two types of flowering, cleistogamic, and chasmogamic, but its reproductive behavior corresponds to that of an autogamous species with a low rate of allogamy (Naranjo, 1992; Gutierrez and Pensiero, 1998). Despite the high autogamy, morphologic and reproductive traits have shown plastic responses to environmental variations, explaining the greater adaptability of *B. catharticus* (Aulicino and Arturi, 2002).

This weed has shown a constancy (i.e., proportion of fields in which a given species is present) of around 20% in winter crops of central Pampas, Argentina (Poggio et al., 2004). In this region, *B. catharticus* starts the life cycle at midsummer and autumn (Ahumada and Troiani, 2016; Iroulart, 2020), when glyphosate is widely used for fallow weed control prior to sowing winter crops such as wheat and barley (Vigna et al., 2014). Biological characteristics, such as genetic plasticity, may predispose a weed species to evolve herbicide resistance (Moss et al., 2019). An herbicide treatment constitutes a challenging environment for a weed population, where an elimination of most local individuals occurs, but the adaptation from standing genetic variation allows the evolution of the population (Matzrafi et al., 2020). This process would be intensified when the weed is subjected to a widespread, persistent, and intense selection pressure with an herbicide (Powles and Yu, 2010).

In general terms, a resistant weed can survive to a normally lethal dose of herbicide by different mechanisms classified as target-site or non-target site resistance. The first category includes amino acid substitutions that affect herbicide interactions at the target enzyme and overexpression of the target site (Gaines et al., 2019). Non-target site mechanisms can be associated with the metabolism of the herbicide or exclusion of the herbicide from the target, either physically with enhanced cuticular and other structural barriers or physiologically with active vacuole sequestration, limited cellular uptake, or a rapid necrosis response (Sammons and Gaines, 2014; Ghanizadeh and Harrington, 2017).

In the south of Buenos Aires province, a population of *B. catharticus* was putatively identified as glyphosate-resistant based on the poor control at recommended doses of glyphosate (960 g ae ha^−1^). As a consequence of the ineffective control in pre-sowing, *B. catharticus* becomes problematic weed species in wheat and barley crops, provoking yield losses of up to 70% (Iroulart, 2020). The aim of this work was to evaluate the magnitude of glyphosate resistance in the offspring of a putative glyphosate-resistant *B. catharticus*, and to determine the mechanisms associated with resistance to glyphosate in this weed species.

## Materials and Methods

### Plant Material

In May 2017, 20 survived individuals of *B. catharticus* were collected from a fallow field (38.71°S and 60.48°W) where glyphosate at 960 g ae ha^−1^ had failed to control *B. catharticus*. In the last 8 years, the crop rotation involved wheat-soybean and barley-soybean, where weed control had been based on recurring applications of glyphosate in fallows and soybean crops, pinoxaden, dicamba, 2,4-D, and metsulfuron in wheat and barley crops.

The collected plants had 5–10 tillers and were dug from the field, taking care not to damage the root in the process. Immediately, the plants were transplanted into 2 L pots filled with soil (25% clay, 10% sand, and 4% organic matter) and placed outdoors in the Chacra Experimental Integrada Barrow (38.31°S and 60.23°W). At the end of the plant life cycle, all plants were harvested and manually threshed. Seeds of glyphosate-susceptible (S) *B. catharticus* were obtained from a population established as weed in the experimental station. Spikes of 20 susceptible plants were collected at random on January 2018. The seeds obtained were stored at room temperature until the beginning of the experiments.

For dose-response and shikimic accumulation assays, the plants were grown in 1 L pots filled with soil (25% clay, 10% sand, and 4% organic matter; one plant per pot) in a greenhouse at 21°C (average temperature) during the autumn season in the Chacra Experimental Integrada Barrow. The plants were irrigated according to water demand and avoiding water excess.

For foliar retention, absorption, translocation, metabolism, and enzyme activity assays, the plants were obtained as below: the seed were germinated in trays (15 cm× 15 cm× 8 cm) with peat moss substrate. The trays were taken to a growth chamber calibrated for 26/18°C day/night, 14 h photoperiod at 850 μmol^−2^ s^−1^ of light intensity, and 60% relative humidity. The seedlings germinated of both populations were transplanted into 250 ml (7 cm× 7 cm× 5 cm) pots (one plant plot^−1^) with 230 g of substrate [soil:peat moss (1:1)]. The plants were taken to the greenhouse and irrigated daily.

### Chemicals

A formulated glyphosate product (60.8% dimethyl amine salt of N-phosphomethyl glycine; Panzer® Gold, Argentina) was used in greenhouse tests and laboratory studies. Glyphosate, in analytical grade (>99%, Sigma-Aldrich, Madrid, Spain), was used to evaluate the biochemical and molecular aspects of glyphosate resistance. ^14^C-glyphosate (glycine-2-^14^C), with a radiochemical purity of 95% and specific activity 273.8 MBq mmol^−1^, was obtained from the Institute of Isotopes Co., Ltd. (Budapest, Hungary).

### Dose-Response Assays

The response of the putative glyphosate-resistant population of *B. catharticus* to glyphosate was compared to a susceptible population using dose-response experiments. A completely randomized design was used in order to test the sensitivity of the plants to glyphosate (60.8% dimethyl amine salt of N-phosphonomethyl glycine; Panzer® Gold, Argentina) doses of 0, 240 (only tested in the S population), 480, 960, 1920, 3,840, and 7,680 [only tested in the glyphosate-resistant (R) population] g ae ha^−1^. The treatments were applied to plants with 2–3 tillers using a laboratory belt sprayer calibrated to deliver 200 L ha^−1^ (distilled water was used as carrier). There were 20 replicates for each herbicide rate, wherein each pot was a sampling unit.

Plant survival was recorded at 21 days after glyphosate treatment. Plants with severe visual injury (wilting, chlorosis of newly emerged leaves, and general browning) were recorded as “dead” plants, while “surviving” plants showed no apparent visual injury. The experiment was repeated twice.

### Shikimic Acid Accumulation in Leaves

An experiment was carried out in order to determine the effects of the different doses of glyphosate on the accumulation of shikimate in leaves. R and S plants with 2–3 tillers were treated with glyphosate (60.8% dimethyl amine salt of N-phosphonomethyl glycine; Panzer® Gold, Argentina) at 0, 480, 960, and 1920 g ae ha^−1^. A completely randomized design was used with five replicates per treatment. At 72 h after treatment (HAT), 0.05 g of fresh weight from the middle third of the youngest fully expanded leaf of each replicate was used for shikimic acid determination, following the methodology described by Perez-Jones et al. (2007). Shikimic acid was quantified with a spectrophotometer (Numak 752 UV-Vis) at 382 nm. The determination of the concentration of shikimic acid was based on a shikimate (3a,4a,5b-trihydroxy-1-cyclohexene-1-carboxylic acid, 99%. Sigma Aldrich, Inc.) standard curve. The experiment was repeated twice.

### Glyphosate Foliar Retention

The methodology used for the foliar retention was described by Palma-Bautista et al. (2020) with some modifications and carried out at the University of Córdoba (Spain). Young plants with 4–6 leaves of R and S *B. catharticus* populations were sprayed with 360 g ae ha^−1^ of glyphosate and 100 mg L^−1^ Na-fluorescein using a laboratory system (SBS-060 De Vries Manufacturing, Hollandale, MN, United States) equipped with 8002 flat fan nozzles delivering 200 L ha^−1^ at 250 kPa at the height of 50 cm from plant level. When plants dried (40–60 min), each shoot tissue was cut at ground level. The tissue was submerged in test tubes with 50 ml of 5 mM NaOH for 30 s to remove the spray solution. The washing solution was recovered in glass flasks. Fluorescein absorbance was determined using a spectrofluorometer (Hitachi F-2500, Tokyo, Japan) with an excitation wavelength of 490 nm and an absorbance wavelength at 510 nm. Then, the plants were wrapped in filter paper and oven dried at 80°C for 48 h and weighed. The experiment was laid out in a completely randomized design with 10 replicates. It was repeated twice, and the results as microliter of sprayed solution retained per g dry weight were combined for analysis.

### ^14^C-Glyphosate Absorption, Translocation, and Visualization

^14^C-glyphosate (glycine-2-^14^C) plus commercial glyphosate solution was applied to R and S *B. catharticus* plants following the methods described by Vázquez-García et al. (2020a,b) and carried out at University of Córdoba (Spain). Plants were treated at the 3–4-leaf stage and there were five repetitions and each experiment was arranged in a completely randomized design.

The second leaf was marked and covered with aluminum foil before spraying the whole plant with 360 g ae ha^−1^ glyphosate, and 30 min later the aluminum foil was removed. The final glyphosate concentration corresponded to 360 g ae ha^−1^ in 200 L ha^−1^, which contained a specific activity of 100,000 dpm μl^−1^ (equivalent to 1.667 kBq μl^−1^). Five plants per population were treated with one drop (1 μl plant^−1^) of the solution on the adaxial surface of the second leaf. After treatment, the plants were maintained in the growth chamber at the growing conditions described in section Plant Material.

The absorbed ^14^C-glyphosate was removed from the treated leaves (at 12, 24, 48, 72, 96, and 120 HAT) by washing three times separately with 1 ml of a water-acetone solution (1:1 v/v) each time. The washing solution was mixed with 2 ml of scintillation liquid (Ultima Gold, Perkin-Elmer, BV BioScience Packard, MA, United States) and analyzed by liquid scintillation spectrometry (LSS) using a scintillation counter (LS 6500, Beckman Coulter Inc., Fullerton, CA, United States) with reading time of 10 min per sample.

After washing, whole plants were removed from the pot and sectioned into treated leaves, the remainder of the shoot and the roots (this plant section was carefully washed with distilled water and excess moisture removed with paper towel). The samples were stored in cellulose cones (Perkin-Elmer, BV BioScience Packard, MA, United States), dried in an oven at 60°C for 96 h, and combusted in a biological oxidizer (Packard Tri Carb 307, Packard Instrument Co., Downers Grove, IL, United States). The CO_2_ released from the combustion was captured in 18 ml of a mix of Carbo-Sorb E and Permafluor (1:1 v/v; Perkin-Elmer, BV BioScience Packard, MA, United States). The radioactivity in dpm of each individual sample was quantified by LSS over a 10 min period per sample. The radioactive values of absorption and translocation of ^14^C were expressed as a percentage of the total ^14^C-herbicide applied and absorbed, respectively.

To visualize the translocation of ^14^C-glyphosate, three plants were treated under the same conditions as in the previous assay. At 96 and 120 HAT, plants were washed individually, fixed on filter paper, and dried at 25°C (room temperature) for 1 week. The plants were pressed for 4 h under a phosphor store film (Storage Phosphor System: Cyclone, Perkin-Elmer Packard BioScience BV, MA, United States) and visualized using a phosphor imager Cyclone (Perkin-Elmer, Packard BioScience BV, MA, United States).

### Glyphosate Metabolism

For this assay, plants were treated with a glyphosate dose of 360 g ae ha^−1^ when they were at the 3 to 4-leaf stage, following the procedure and equipment used in the glyphosate foliar retention assay. The same numbers of plants, without glyphosate treatment, were used as blank. Plants were cut at 120 HAT, washed with distilled water (to remove excess herbicide on the surface of the leaf), and dried. Rapidly, they were frozen by liquid nitrogen and stored at a temperature less than or equal to −40°C before being used. For the determination and quantification of glyphosate and its metabolites metabolites [amino methyl phosphonic acid (AMPA), glyoxylate, sarcosine, and formaldehyde], the methodology described by Rojano-Delgado et al. (2010) was followed, using a 3D Capillary Electrophoresis Agilent G1600A instrument equipped with a diode array detector (DAD, wavelength range 190–600 nm). The used background electrolyte was an aqueous solution at pH 7.5, containing 10 mM potassium phthalate, 0.5 mM hexadecyltrimethylammonium bromide (CTAB), and 10% acetonitrile. The calibration equations were obtained using standards of known concentration of glyphosate and metabolites were supplied by Sigma-Aldrich (St. Louis, MI). The experiment was arranged in a completely randomized design with five replications (individual plants) per population and treatment and repeated three times.

### EPSPS Basal Activity and Dose-Response

Five grams of leaf tissue from R and S plants, finely powdered, were transferred to tubes with 100 ml of cold extraction buffer (100 mM MOPS, 5 mM EDTA, 10% glycerol, 50 mM KCl, and 0.5 mM benzamidine), 70 μl of β-mercaptoethanol and 1% polyvinylpolypyrrolidone (PVPP). Enzyme extraction was performed following the protocol described by Sammons et al. (2007).

The specific 5-enolpyruvylshikimate-3-phosphate synthase (EPSPS) activity was assayed in the presence of glyphosate (>99%) at different concentrations (from 0 to 5,000 μM) using the EnzChek Phosphate Assay Kit (Invitrogen, Carlsbad, CA, United States). The EPSPS enzyme reaction substrates were phosphoenolpyruvate and shikimate-3-phosphate, which were supplied by Sigma-Aldrich (Madrid, Spain). The release of phosphate was measured for 10 min at 360 nm in a spectrophotometer (model DU-640, Beckman Instruments Inc., Fullerton, United States). The total soluble protein (TSP) in the extract was measured using a Kit for Protein Determination (Sigma-Aldrich, Madrid, Spain), following the manufacturer’s instructions. The EPSPS activity was measured for 10 min at 360 nm in a spectrophotometer (model DU-640) to determine the amount of inorganic phosphate (μmol) released per μg of TSP per min (μmol Pi μg^−1^ TSP min^−1^). The EPSPS activity was expressed as a percentage relative to the control (absence of glyphosate). Three technical replications of each glyphosate concentration were analyzed per population. The experiment was repeated twice.

### *EPSPS* Gene Sequencing

Total DNA was extracted from the leaf tissue of five R plants (survivors at a glyphosate dose of 1,920 g ae ha^−1^), following the protocol of Doyle and Doyle (1990). DNA yield and quality were evaluated spectrophotometrically. The DNA was used as a template to amplify the EPSPS sequence. The forward primer (5'-AGCTGTAGTCGTTGGCTGTG-3') and reverse primer (5'-GCCAAGAAATAGCTCGCACT-3') were employed to amplify a highly conserved region encompassing the positions of all the known mutations that confer glyphosate resistance (Sammons and Gaines, 2014). A 1,395-bp fragment was obtained in the PCR reactions (initial denaturation at 94°C for 2 min and 30 cycles of 94°C for 1 min, 62°C for 1 min, 72°C for 1 min and final extension at 72°C for 5 min), containing: 300 ng DNA template, 0.8 μM of each primer, 0.2 mM of each dNTPs, 1.5 mM MgCl_2_, 1X reaction buffer (Inbio Highway), and 1 U Taq polymerase (Inbio Highway) in a 25 μl reaction mix.

PCR products were purified and sequenced from both ends through Macrogen service (Macrogen Inc., Seoul, South Korea). The sequence data obtained were cleaned, aligned, and compared at 101, 102, 106, 144, and 192 codons (numbers based on the plant EPSPS numbering system used by Padgette et al., 1996) using BLAST of the National Center for Biotechnology Information (NCBI).

### Statistical Analysis

Survival and EPSPS activity data were used to build dose-response curves with a non-linear log-logistic regression model as described by Streibig et al. (1993):

$$y=c+\{\left(d-c\right)/\left[1+{\left(x/g\right)}^{b}\right]\}$$

In this equation, *y* represents the percentage of response at the herbicide rate *x*; *c* and *d* are the lower (fixed at 0 for LD_50_) and upper asymptote, respectively; *b* is the slope of the line at *g*; and *g* is the herbicide concentration required to reach 50% of the maximum response for EPSPS enzyme activity (I_50_) or the glyphosate dose causing 50% mortality (LD_50_). To assess the accuracy of the models, F-test for model significance, residual variance analysis, and coefficient of determination (R^2^) were calculated. LD_50_ and I_50_ values from resistant and susceptible populations were compared with the F-test (*p* < 0.05; GraphPad Prism 6 Software) and a resistance index (RI) was calculated as the ratio of the LD_50_ of the resistant population compared to the susceptible population.

An ANOVA was performed to evaluate the differences between populations and treatments. The differences between the mean values of shikimic acid contents, glyphosate foliar retention, and ^14^C-glyphosate absorption and translocation were compared with Fisher test (*p* < 0.05; Statistica® v7.1. Stat Soft).

## Results

### Glyphosate-Sensitivity: Plant Survival and Shikimate Accumulation

**Figure 1:**
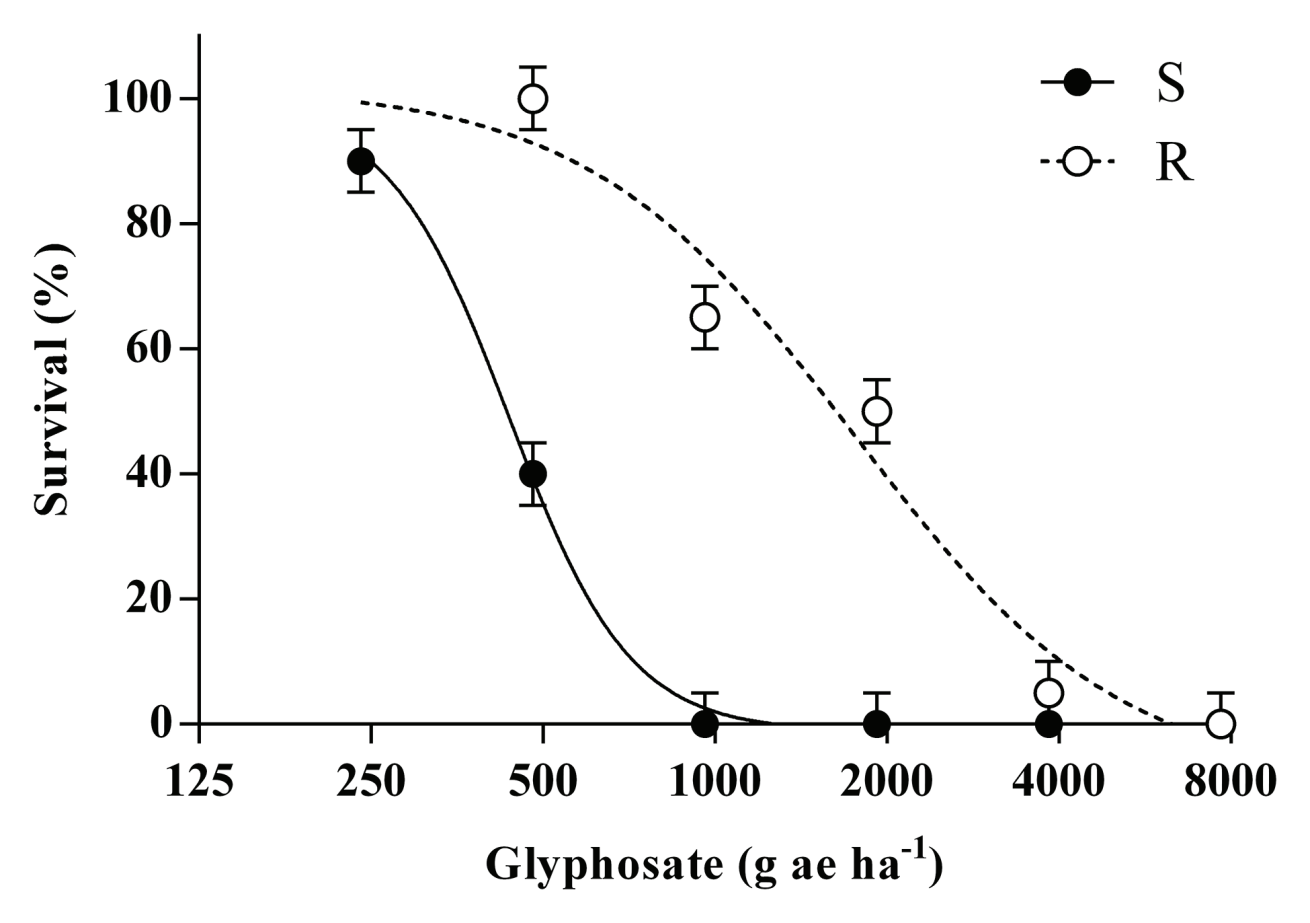
Effects of glyphosate doses on plant survival for the glyphosate-susceptible (S) and -resistant (R) *Bromus catharticus* at 21 days after treatment. Symbols represent mean values and bars indicate ± 1 SEM. The predicted responses are shown by lines according to the adjusted models: (S) y = −1+(99+1)/[1+(x/436)^4.1^]; *p* < 0.01; R^2^ = 0.99 and (R) y = −9+(102+9)/[1+(x/1750)^1.8^]; *p* < 0.01; R^2^ = .97.

At least 50% of plants from the R population survived to 1 and 2-fold of the recommended dose of glyphosate (960 g ae ha^−1^); while none of the S plants survived these treatments (Figure 1). Regression models fitted to plant survival of both populations were compared, and LD_50_ parameters differed significantly between S and R populations (*p* = 0.004). The LD_50_ calculated for the R population was higher than the recommended dose of glyphosate (1750 vs. 960 g ae ha^−1^) and the RI achieved was 4.0.

**Figure 2:**
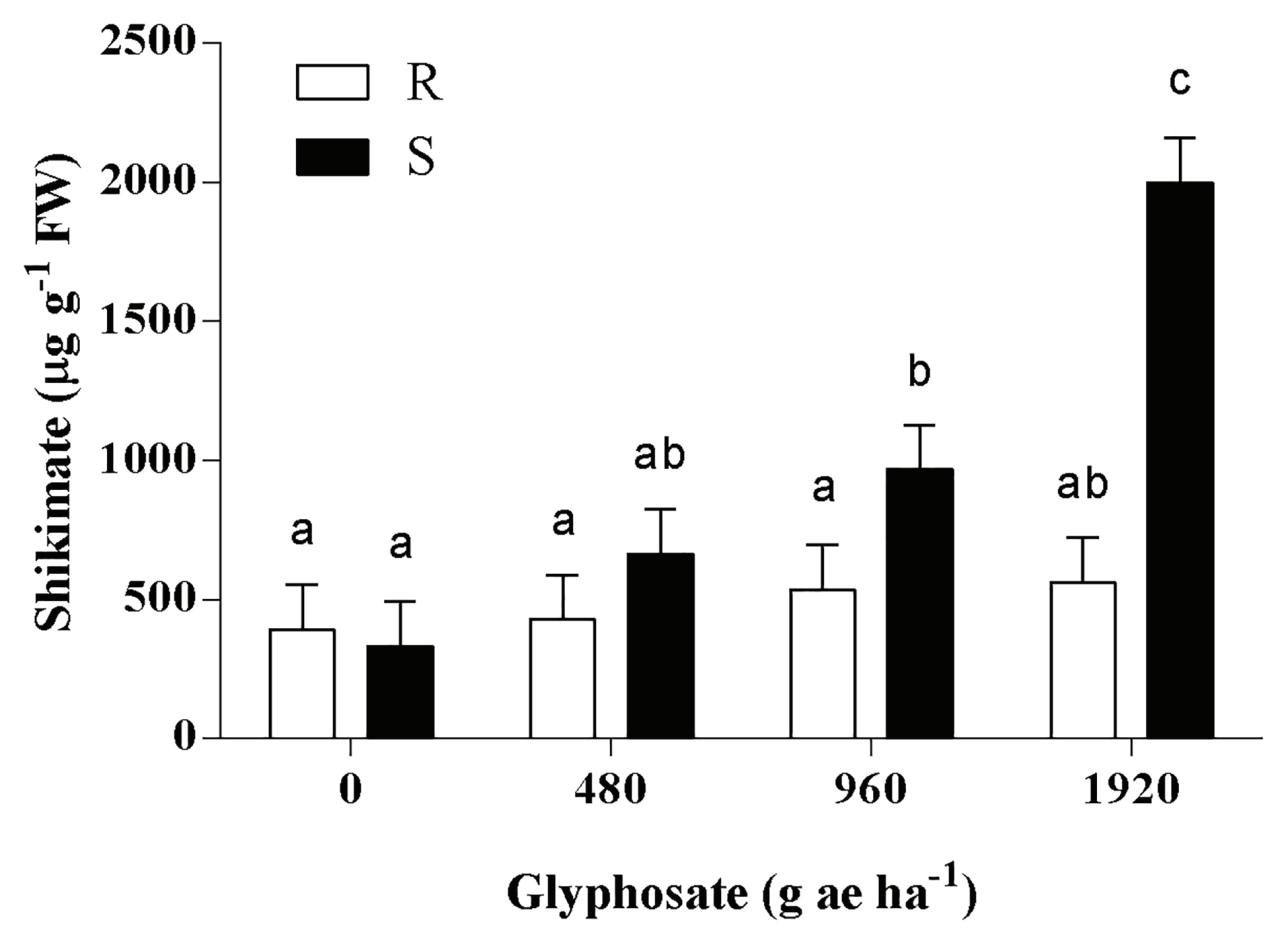
Accumulation of shikimic acid in the last expanded leaf of glyphosate-susceptible (S) and -resistant (R) *Bromus catharticus* at 72 h after glyphosate treatment. Columns represent mean values, and vertical bars indicate the SEM. Letters above the bars indicate statistical significance (*p* < 0.05).

The response of shikimate accumulation to the glyphosate dose was significantly different between populations (*p* < 0.01; Figure 2). While the basal content of shikimate was similar in S and R plants, a significant shikimate accumulation of 2.9 and 6.0-fold was detected in S plants when treated with 960 and 1920 g ae ha^−1^, respectively. In contrast, R plants showed no significant changes in shikimate concentration among the different treatments (Figure 2).

### Glyphosate Foliar Retention

**Figure 3:**
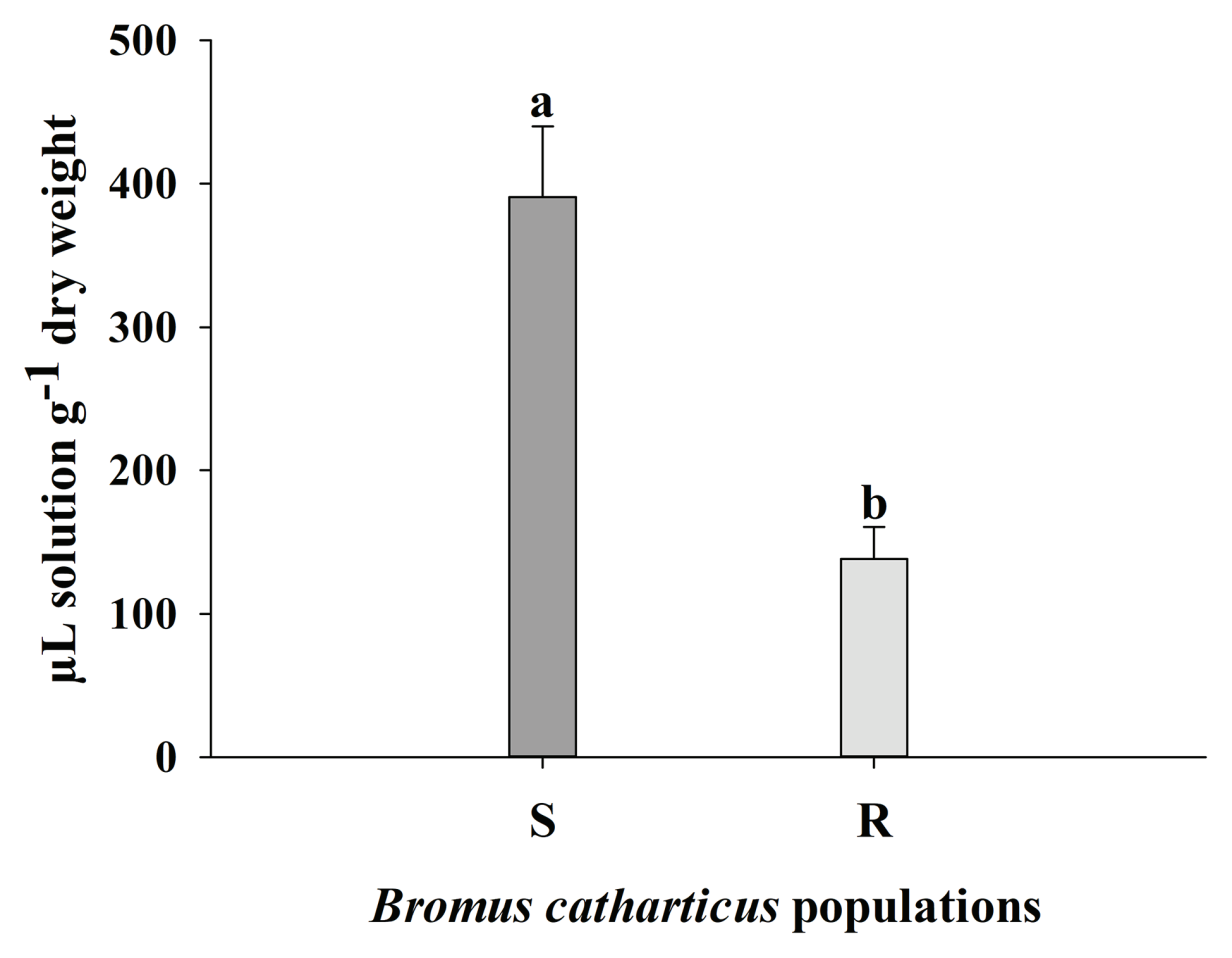
Spray retention of a glyphosate solution by resistant (R) and susceptible (S) *Bromus catharticus* plants. Error bars are the SEM. Different letters above the bars indicate significant differences between populations (*p* < 0.05).

S and R plants of *B. catharticus* treated with glyphosate showed different foliar retention of the herbicide. S leaf retention was 390.79 ± 49.10 (SE; μl solution g^−1^ dry weight) while the R population had a lower value of 138.34 ± 22.36 (SE; μl solution g^−1^ dry weight). Foliar retention capacity was 2.83 times greater in the S population as compared to the R population (Figure 3).

### ^14^C-Glyphosate Absorption, Translocation, and Visualization

**Figure 4:**
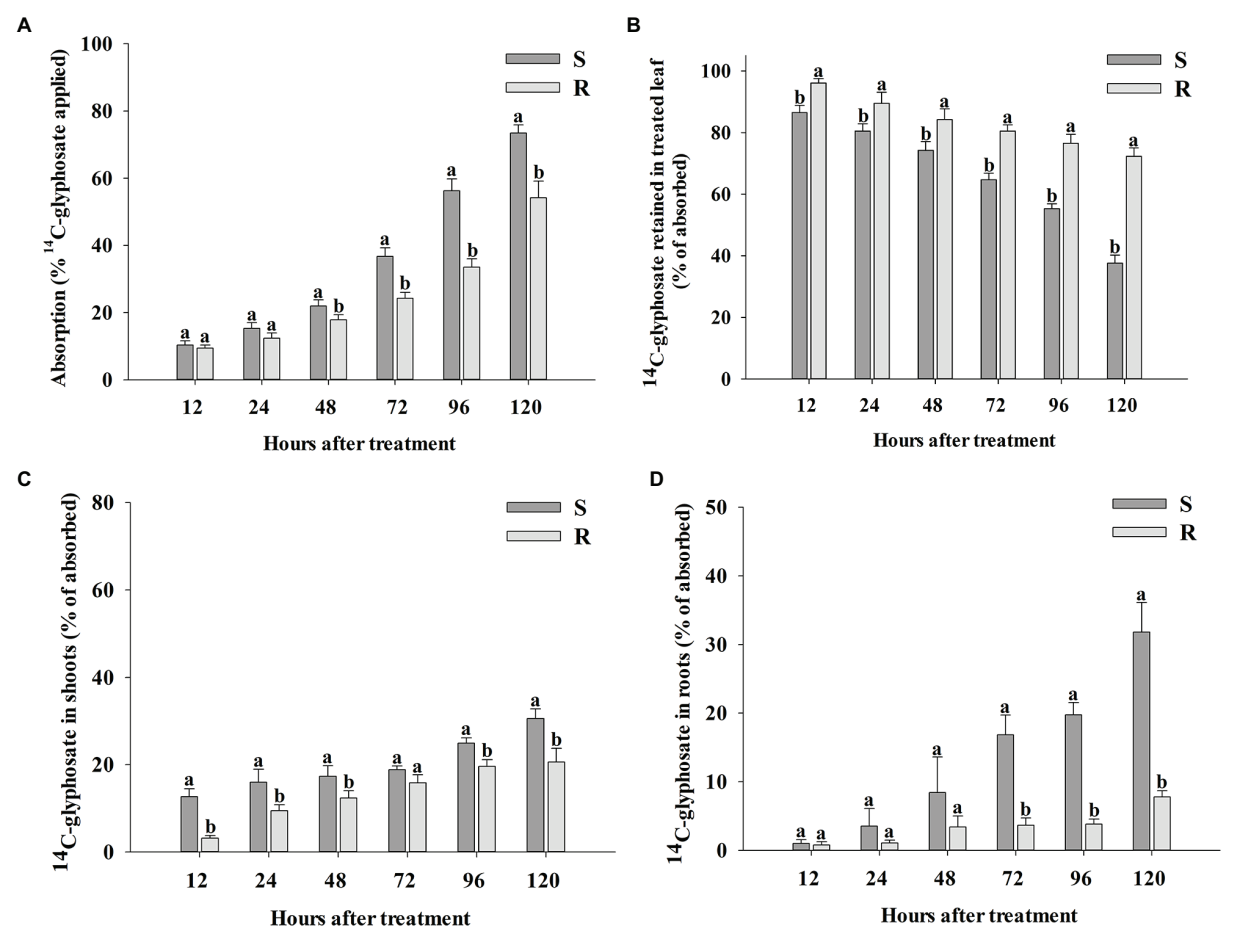
Percentage of absorption of ^14^C-glyphosate **(A)** in glyphosate-susceptible (S) and -resistant (R) *Bromus catharticus*, ^14^C-glyphosate detected in the labeled leaf **(B)** and translocation from treated leaf to rest of plants **(C)** and the root system **(D)** at 12–120 HAT. Error bars are the SEM per time evaluated. Different letters above the bars indicate statistical differences between populations at the same time of evaluation (*p* < 0.05).

Total recovery of ^14^C-glyphosate in this research was 94.3 ± 2.1 (SE) % and 95.1 ± 1.4 (SE) % for R and S populations of *B. catharticus*, respectively. Absorption of ^14^C-glyphosate was slow in both R and S populations until 48 HAT (Figure 4A). At this time, the S population had absorbed 21.96 ± 1.81 (SE) % glyphosate, while the R population had only absorbed 17.86 ± 1.47 (SE) %. From 48 HAT, the absorption began to be exponentially more pronounced in the S population. The maximum absorption rate of glyphosate was observed at 120 HAT, which was 1.4-fold higher in the S population [73.56 ± 2.40 (SE) %] than in the R population [54.18 ± 4.94 (SE) %]. At any time, compared with the R plants, S plants translocated more ^14^C-glyphosate (as a percentage of that absorbed) from the treated leaves to the rest of the plant and roots (Figures 4B–D). The corresponding accumulation of ^14^C-glyphosate was measured in the remaining shoot tissue (rest of plants) was greater for the S population as compared to R counterparts (Figure 4C). Differences in accumulation of ^14^C-glyphosate in roots between S and R populations were most noticeable at times later than 48 HAT. The S vs. R plants demonstrated a 4.1-fold higher root concentration of ^14^C-glyphosate at 120 HAT (Figure 4D).

**Figure 5:**
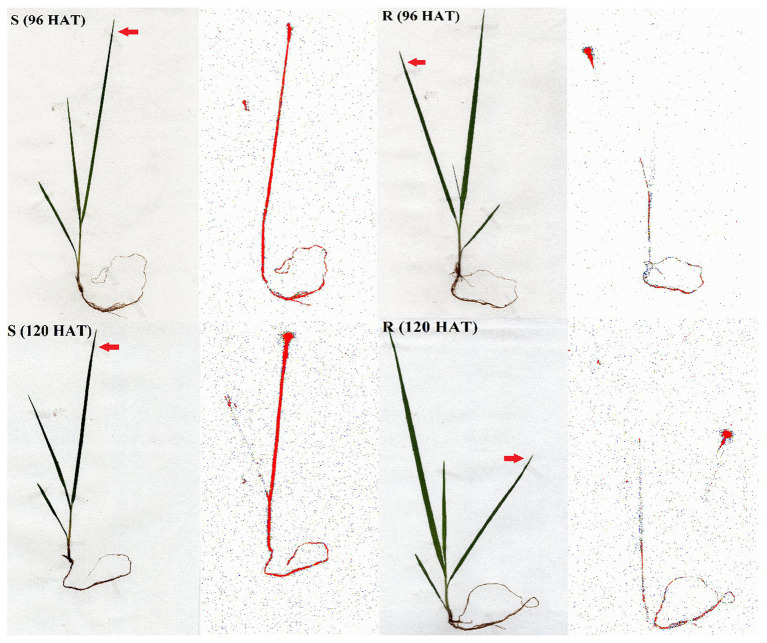
Digital images and ^14^C-glyphosate visualization in glyphosate-susceptible (S) and -resistant (R) *Bromus catharticus* populations. The translocation visualization was obtained from treated plants at 96 and 120 HAT. Arrows indicate the treated leaves. The concentration of ^14^C-glyphosate is highlighted in red.

The Phosphor Imager images shown confirmed the previous results obtained with the LSS in absorption and translocation assays (Figure 5). At 96 and 120 HAT, the plants of the R population absorbed and translocated smaller amounts of ^14^C-glyphosate from the treated leaf to the root than the S plants (Figure 4).

### Glyphosate Metabolism

The data obtained in this study (Table 1) showed that there is no glyphosate metabolism. In fact, only glyphosate and a minimum amount of glyoxylate can be observed at 120 HAT. The latter cannot be considered a metabolite of glyphosate because its origin is not only glyphosate; therefore, if AMPA does not appear (for example), it cannot be considered as a metabolite of this herbicide.

**Table 1:** Glyphosate metabolism (%) at 120 h after treatment (HAT) in glyphosate-susceptible (S) and -resistant (R) *Bromus catharticus* populations. Mean values ± SE are shown.

Metabolism (%) at 120 HAT			
*B. catharticus*	Glyphosate	AMPA	Glyoxylate
S	97.48 ± 3.18	–	2.81 ± 0.82
R	96.92 ± 2.21	–	3.05 ± 0.49

### EPSPS Basal Activity and I_50_ Values

**Figure 6:**
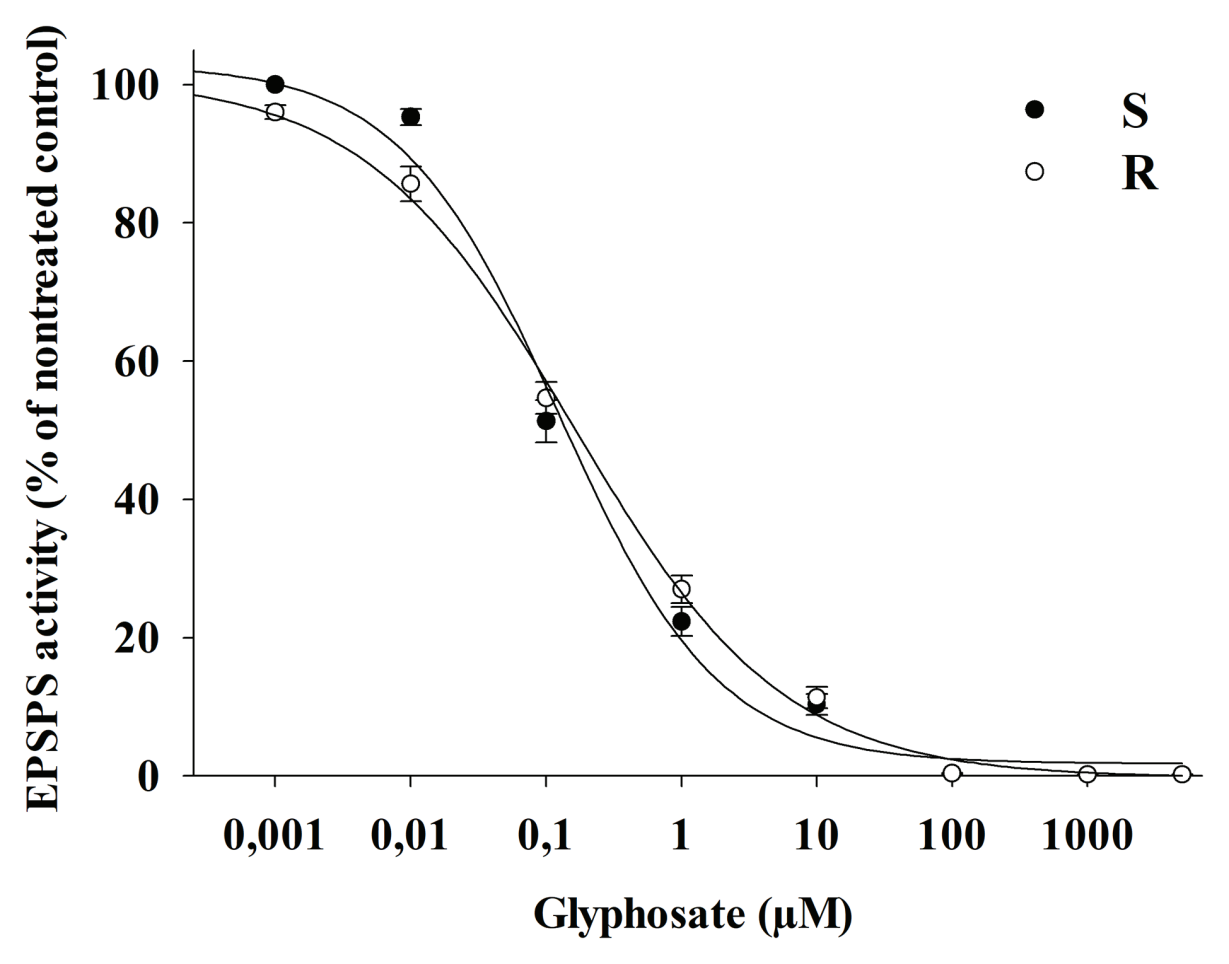
EPSPS enzyme activity of glyphosate-susceptible (S) and -resistant (R) *Bromus catharticus* populations exposed to different glyphosate concentrations (μM), expressed as a percentage of the untreated control. The predicted responses are shown by lines according to the adjusted models: (S) y = (103)/[1+(x/0.12)^0.73^]; *p* < 0.01; R^2^ = 0.99 and (R) y = (101)/[1+(x/0.16)^0.56^]; *p* < 0.01; R^2^ = 0.99.

No differences were observed between R and S populations in respect to the concentration of glyphosate required to inhibit EPSPS activity by 50% (I_50_), being 0.150 and 0.120 μM of glyphosate, respectively (Figure 6). In addition, the EPSPS activity in the absence of glyphosate was similar in both R and S populations, being 1.82 ± 0.03 (SE) and 1.45 ± 0.03 (SE) μmol Pi μg^−1^ TSP min^−1^, respectively. In this case, no differences were apparent between the S and R plants for either EPSPS activity in the absence of glyphosate or the inhibition response to glyphosate (I_50_).

### *EPSPS* Gene Sequencing

The fragment sequenced (MT454262) included exons (part of 2, 3, and 4) and introns (2, 3, and part of 4) according to the structure described by Aramrak et al. (2015) The sequence of *B. catharticus* showed around 86% of identity with the three genomic copies of *EPSPS* of *T. aestivum* on chromosomes 4A, 7A, and 7D (KP411547.1, KP411548.1, and KP411549.1). However, the exons were more highly conserved than introns, where the translated regions showed at least 90% of identity with the three genes of *T. aestivum*.

Analyzing the differences between exons of *B. catharticus* vs. *T. aestivum*, most nucleotide mismatches were associated with silent substitutions, but seven mismatches involved six codon changes: Gly-134-Ala, Gln-141-Lys, Asp-146-Thr, Asn-154-Asp, Lys-163-Thr, and Glu-194-Gly. However, no substitutions were recorded at codons 101, 102, 103, 106, 144, and 192, which have been associated with low glyphosate-sensitivity.

### Discussion

Within the genus *Bromus*, populations of *B. diandrus*, *B. sterilis*, and *B. rubens* have evolved glyphosate resistance in Australia, United Kingdom, and Spain, respectively (Malone et al., 2016; Davies et al., 2019; Heap, 2020), and *EPSPS* gene amplification was reported as the mechanism of glyphosate resistance in *B. diandrus* (Malone et al., 2016). Until now, no antecedents of glyphosate-resistance were detected in American species of *Bromus*.

In the current work, the plant survival analysis showed that the glyphosate-sensitivity of the R *B. catharticus* population was around 4-fold lower than the S population (Figure 1). Consistent with this finding, the shikimate content in the leaves of R plants showed no significant changes in response to glyphosate treatments (Figure 2). These results support an inherited ability of plants from the R population to survive and reproduce after a normally lethal dose of glyphosate. All surviving plants produced viable seeds, so in that sense, the progeny obtained from individuals treated to 1,920 g ae ha^−1^ was used to determine the mechanism of resistance.

No evidence was found for target-site mechanisms of resistance to glyphosate in the R population. Neither a target-site mutation nor differences in basal EPSPS activity were identified between both populations. However, our results revealed that R plants retained just under half of the amount quantified in S plants (Figure 3) but spraying a double dose of glyphosate on the R population was not enough to match the mortality of the S population (Figure 1). A lower foliar retention of herbicide would constitute the first barrier for glyphosate efficacy because it limits the amount that can subsequently enter the plant. In any case, a differential glyphosate foliar retention has not been detected as a strong mechanism of resistance *per se*, which instead has been linked to an altered glyphosate uptake and translocation or even duplication of *EPSPS* gene copies in glyphosate-resistant *Lolium multiflorum* populations (Michitte et al., 2007; Fernández-Moreno et al., 2017). In the *B. catharticus*, population analyzed important mechanism of herbicide selectivity would be associated with other exclusion mechanisms as in the cases quoted above.

No evidence was obtained for an enhanced capacity to metabolize glyphosate during the time of evaluation (up to 120 HAT; Table 1), and this observation supports the tracking of glyphosate movement in order to confirm differences in herbicide uptake and translocation between populations. Comparing the glyphosate absorption process in both populations, R plants showed a slower uptake from 48 to 120 HAT. Thus, the maximum absorption rate of glyphosate was detected at 120 HAT, which was 25% lower in the R population in respect to the glyphosate uptake quantified in S plants (Figure 4A). A differential rate of glyphosate entry into leaves has been detected in other weed species, where R accessions showed up to 40% of reduction in the absorption of herbicide; however, this trait seems to be frequently associated with an altered pattern translocation (Michitte et al., 2007; de Carvalho et al., 2012; Vila-Aiub et al., 2012; Dominguez-Valenzuela et al., 2017; Palma-Bautista et al., 2019).

Notwithstanding differences in glyphosate absorption between populations, the current results show that herbicide uptake was around 10–15% of glyphosate applied at 12 and 24 HAT and no differences were detected between R and S plants during this period (Figure 4A). Nevertheless, the glyphosate translocation from the labeled leaf was significantly different between both populations at 12 and 24 HAT (Figures 4B,C). Throughout the entire analysis period, R plants translocate half or less of the glyphosate absorbed compared to S plants (Figure 4B). As a consequence, the translocation of glyphosate to the roots was four times greater in S plants as compared to R counterparts at the last moment of evaluation (Figure 4D). This evidence suggests that impaired translocation of glyphosate would be the primary mechanism of resistance in the R *B. catharticus* population.

The reduction in glyphosate movement to sensitive tissues, such as shoots and root meristems, would have a large effect on plant survival (Preston and Wakelin, 2008; Shaner, 2009). Since the herbicide affects actively growing tissues, the demand for assimilates would decrease and consequently induce an accumulation of carbohydrates in the leaves associated with a feedback inhibition of CO_2_ fixation, but as the light stage of photosynthesis is initially unaffected, a redirection of electrons to alternative electron sinks occurs, conducive to oxidative stress, that ultimately leads to plant death (Yanniccari et al., 2012a,b). As a consequence, a restricted translocation of glyphosate has been frequently detected as an important mechanism of resistance in several weed species (Feng et al., 2004; Wakelin et al., 2004; Koger and Reddy, 2005; Vila-Aiub et al., 2012; Ghanizadeh et al., 2016; Dominguez-Valenzuela et al., 2017; Vázquez-García et al., 2020a,b). Going further, a process of glyphosate sequestration within the cell vacuole was detected as a basis of glyphosate altered translocation in resistant *Conyza canadensis* and *Lolium* spp. populations (Ge et al., 2010, 2012, 2014).

*Bromus* spp. weeds have emerged as a major challenge for arable farmers because there is no effective herbicide for its control in cereals (Dastgheib et al., 2003). In that sense, *B. catharticus* should be controlled during the fallow period prior to sowing winter cereals, for which glyphosate is widely used in Argentina (Vigna et al., 2014). The current work shows the first worldwide case of glyphosate resistance in *B. catharticus*, where an altered glyphosate translocation pattern was revealed from 12 HAT onwards and this represented to be the primarily mechanism of resistance. However, a lower foliar retention and a reduced absorption of glyphosate were also evidenced as barriers of glyphosate exclusion mechanisms. Given the relevance of genetic factors in the dynamics of herbicide-resistance (Ghanizadeh et al., 2019), it is important to know the basis of the inheritance of glyphosate resistance in *B. catharticus*. Preventing the spread of this trait is a major challenge for future studies, considering the high capacity of adaptation of this weed to a wide range of habitats and growing conditions.

## Data Availability Statement

The datasets presented in this study can be found in online repositories. The names of the repository/repositories and accession number(s) can be found at: https://www.ncbi.nlm.nih.gov/genbank/, MT454262.

## Author Contributions

MY, JV-G, PA, and RP designed experiments. MY, JV-G, AR-D, and MG-L performed the experiments and data analysis. MY, JV-G, AR-D, PA, and RP wrote the manuscript. All authors have reviewed and approved the final manuscript.

### Conflict of Interest

The authors declare that the research was conducted in the absence of any commercial or financial relationships that could be construed as a potential conflict of interest.

The handling editor declared a shared affiliation with several of the authors, MY and MG-L, at the time of the review.
